# Abiotic environmental factors override phytoplankton succession in shaping both free-living and attached bacterial communities in a highland lake

**DOI:** 10.1186/s13568-019-0889-z

**Published:** 2019-10-31

**Authors:** Huan Wang, Rong Zhu, Xiaolin Zhang, Yun Li, Leyi Ni, Ping Xie, Hong Shen

**Affiliations:** 1 Donghu Experimental Station of Lake Ecosystems, State Key Laboratory of Freshwater Ecology and Biotechnology of China, Institute of Hydrobiology, Chinese Academy of Sciences, Wuhan, 430072 People’s Republic of China; 2 Nanjing Institute of Geography & Limnology, Chinese Academy of Science, Nanjing, 210008 China; 3 State Key Laboratory of Plateau Ecology and Agriculture, Qinghai University, Xining, 810016 China

**Keywords:** Abiotic environmental factors, Attached bacteria, Free-living bacteria, Phytoplankton community, Variation partitioning in redundancy analysis

## Abstract

Bacterial communities are an important part of biological diversity and biogeochemical cycling in aquatic ecosystems. In this study, the relationship amongst the phytoplankton species composition and abiotic environmental factors on seasonal changes in the community composition of free-living and attached bacteria in Lake Erhai were studied. Using Illumina high-throughput sequencing, we found that the impact of environmental factors on both the free-living and attached bacterial community composition was greater than that of the phytoplankton community, amongst which total phosphorus, Secchi disk, water temperature, dissolved oxygen and conductivity strongly influenced bacterial community composition. *Microcystis* blooms associated with subdominant *Psephonema* occurred during the summer and autumn, and *Fragilaria*, *Melosira* and *Mougeotia* were found at high densities in the other seasons. Only small numbers of algal species-specific bacteria, including *Xanthomonadaceae* (*Proteobacteria*) and *Alcaligenaceae* (*Betaproteobacteria*), were tightly coupled to *Microcystis* and *Psephonema* during *Microcystis* blooms. Redundancy analysis showed that although the composition of the bacterial communities was controlled by species composition mediated by changes in phytoplankton communities and abiotic environmental factors, the impact of the abiotic environment on both free-living and attached bacterial community compositions were greater than the impact of the phytoplankton community. These results suggest that the species composition of both free-living and attached bacterial communities are affected by abiotic environmental factors, even when under strong control by biotic factors, particularly dominant genera of *Microcystis* and *Psephonema* during algal blooms.

## Introduction

Bacterial communities adapt to environmental changes due to their small size, short biological life cycles and genetic variability (Lenski 2017; McAdams et al. 2004). Moreover, the species composition of bacterial communities is a robust indicator of ecological dynamics in aquatic ecosystems (Glasl et al. 2017: Harnisz 2013; Karimi et al. 2017). The Baas Becking in the widely referenced quote, “everything is everywhere, but environments select” (De Wit and Bouvier 2006) assumes a common existence of microorganisms based on high dispersion ratios. Following the widespread application of molecular biological techniques, studies on the species composition of bacterial communities have shaped by both biotic communities (Berry et al. 2017; Camarena-Gómez et al. 2018) and abiotic environmental factors (Fraser et al. 2018; Scofield et al. 2015). For example, in lakes, both phytoplankton biomass (Luria et al. 2017) and physicochemical environmental conditions (Adamovich et al. 2019; Liu et al. 2015) influence the species composition of bacterial communities. Understanding the response of bacterial communities to the seasonal changes of the abiotic environment, including changes in the phytoplankton species composition, are necessary to understand changes in species composition or the functional roles of the bacterial communities in lakes (Gilbert et al. 2012; Luria et al. 2017; Paver and Kent 2017; Yannarell and Triplett 2005).

Abiotic environmental factors, such as water temperature (Paver and Kent 2017; Yannarell and Triplett 2005), pH (Liu et al. 2015), total nitrogen (Tian et al. 2009), total phosphorus (Fraser et al. 2018; Romina Schiaffino et al. 2011), salinity (Fraser et al. 2018; Kirchman et al. 2017; Wu et al. 2006) and dissolved oxygen (Tian et al. 2009) affect the species composition of bacterial communities in lakes as many bacterial taxa exhibit specific environmental preferences. Moreover, the phytoplankton community acts as a biotic influence and interacts with the bacterial community through direct or indirect interactions including mutualism, commensalism, parasitism, amensalism and competition (Kazamia et al. 2016; Kirchman et al. 2017; Landa et al. 2016; Lovdal et al. 2007; Seymour et al. 2017; Sison-Mangus et al. 2016). For example, phytoplankton provide habitats and species-specific exudates for some bacterial species (Paver et al. 2013; Sapp et al. 2007; Seymour et al. 2017) and bacteria can support the growth of phytoplankton via nutrient recycling (Harte and Kinzig 1993). Competition between phytoplankton and bacterial communities are regarded as important biological interactions controlled by limiting nutrients (Currie and Kalff 1984; Danger et al. 2007), allelopathic chemicals (Cole 1982) and other physiological traits (Rooney-Varga et al. 2005). Therefore, bacterial communities are important components of lakes, and their species composition is affected by abiotic and biotic factors, particularly the phytoplankton community (Goecke et al. 2013; Tujula et al. 2010).

More specifically, bacterial communities in lakes can be divided into two components, attached and free-living bacteria (Tang et al. 2017). Attached bacteria (particles ≥ 5 μm in diameter that are retained by a glass fiber filter) and free-living bacteria (that pass through the filter) are considered separate populations (Niu et al. 2011; Rooney-Varga et al. 2005). Attached bacteria have been found growing on the surface of algal cells and show symbiotic associations with algae (Seymour et al. 2017); free-living bacteria are less closely associated with algal cells, particularly during algal blooms when extremely high cell densities occur (Sison-Mangus et al. 2016). For bacteria attached to phytoplankton, the phytoplankton provide distinct metabolic functions and enzymes (Bagatini et al. 2014). Functionally similar bacterial species are often attached to similar algal taxa or groups (Rooney-Varga et al. 2005; Sapp et al. 2007). However, it remains to be elucidated how abiotic environmental factors and phytoplankton species composition interactively influence attached and free-living bacterial communities.

To address this issue, we investigated the seasonal variation in the species composition of free-living and attached bacteria over an 18-month period, and analyzed the response of the community composition of free-living and attached bacteria to changes in both phytoplankton composition and abiotic environmental factors in Lake Erhai. Three hypotheses were assessed: (1) based on the findings of previous studies that showed the importance of abiotic environmental factors and that phytoplankton succession occurs between attached and free-living bacteria (Sapp et al. 2007; Tang et al. 2017), we assessed changes in the species composition of free-living and attached bacterial communities according to phytoplankton species composition and abiotic environmental factors; (2) although bacterial communities are impacted by the coupled effects of changes in phytoplankton species composition and abiotic factors (Niu et al. 2011; Paver and Kent 2017), we assessed whether abiotic environmental variations are dominant factors in shaping bacterial community composition; and (3) since phytoplankton provide habitats and exudates for some bacterial species (Paver et al. 2013; Sapp et al. 2007; Seymour et al. 2017), we tested whether the impact of changes in phytoplankton species composition on the attached bacterial communities are greater than free-living bacterial community. Understanding the effects of changes in phytoplankton species composition and abiotic factors on the seasonal patterns of bacterial species composition will provide important insight into the factors controlling species composition of the bacterial communities in lakes, and will improve our ability to predict the bacterial response to both abiotic and biotic environmental changes.

## Methods

### Study zones and site description

**Fig. 1:**
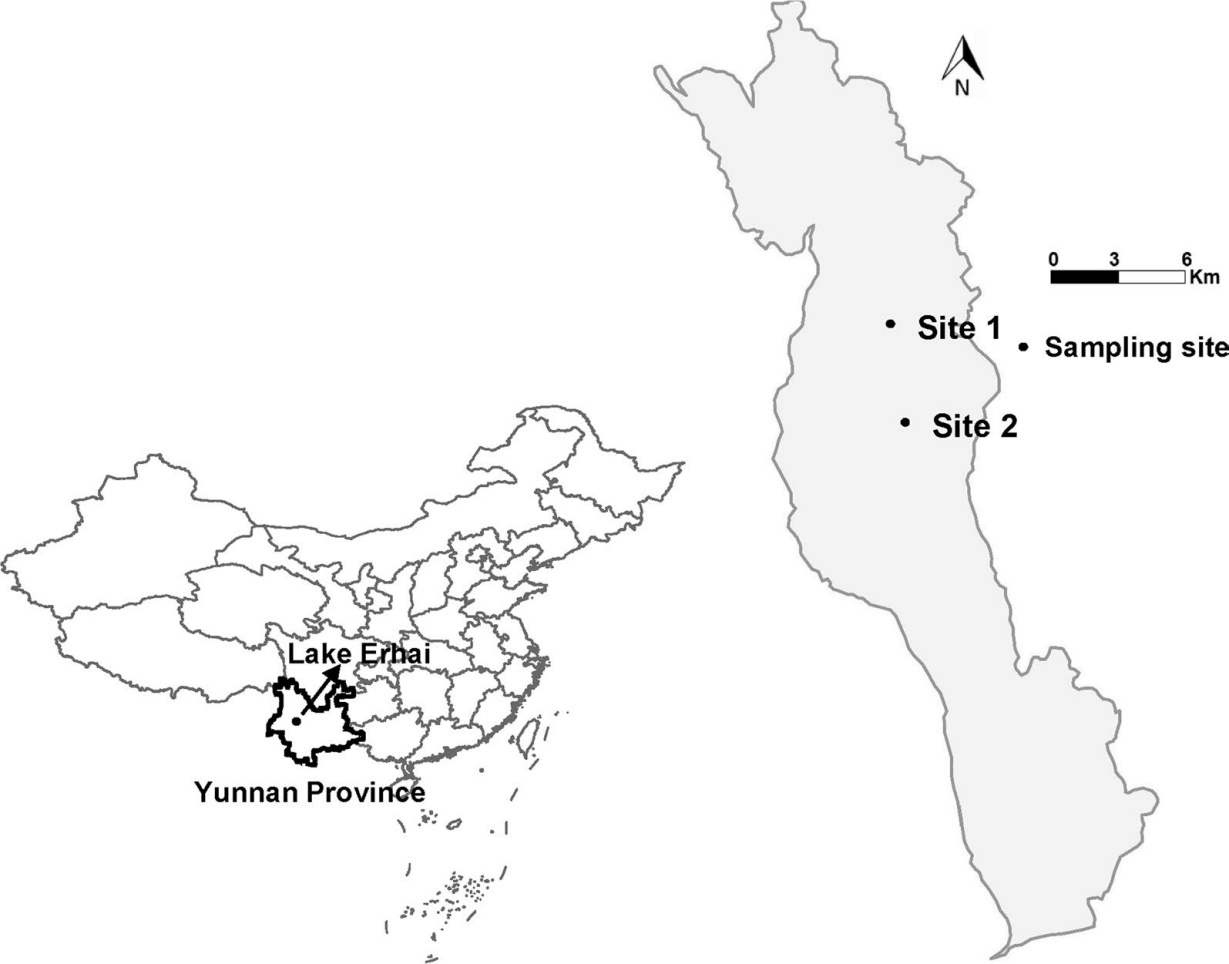
A map of Lake Erhai with the locations of the sampling sites

This study was performed in Lake Erhai (25°36′–25°58′N, 100°05′–100°17′E), the second largest high-altitude freshwater lake on the Yunnan Plateau, in the central zone of the Dali Bai Autonomous Prefecture in Yunnan Province, China (Fig. 1). The lake has a total surface area of ~ 250 km^2^, an elevation of 1974 m and a volume of nearly 28.8 × 108 m^3^. The average and maximum depths are 10.5 m and 20.5 m, respectively. Our published study show that the total nitrogen and total phosphorus levels of Lake Erhai are approximately 0.7 mg/L and 0.03 mg/L, respectively (Zhu et al. 2018). Lake Erhai is now in the early stage of eutrophication. Prior to the 1970s, Lake Erhai was an oligotrophic lake (Jin et al. 2005). Since the 1980s, the Erhai Lake has been affected by man-made eutrophication with an increasing population of residents (Wang et al. 2012). After the 1990s, as the district population continued to increase and human activities grew, the ecological environment deteriorated and nutrient loading increased, resulted in the frequent occurrence of cyanobacterial blooms (Tan et al. 2017). Five large blooms occurred in the lakes in 1998, 2003, 2006, 2009, and 2013 (Zhu et al. 2018).

Sampling was performed monthly from May 2013 to October 2014 at two sampling sites (site 1, 25°50′7.78″N, 100°11′31.48″E; site 2, 25°47′59.49″N, 100°11′49.25″E) in the central lake region (Fig. 1). Based on field surveys in Lake Erhai performed from 2013 to 2016 (unpublished data), the patterns in algal succession regarding the dominant phytoplankton species showed comparable patterns in different areas within the pelagic zone. We were therefore confident that our sampling sites accurately represented the dynamics of the pelagic zone. A composite sample was collected by combining water samples from the upper (0.5 m below the water surface), middle (midway between the surface and the bottom: site 1: 7 m; site 2: 3.5 m), and lower (0.5 m above the sediment surface: site 1: 14 m; site 2: 7 m) portions of the water column at each site. The composite samples were used to analyze hydrochemical parameters and phytoplankton composition. Samples (200 to 500 mL) for bacterial analysis were collected on 0.2 μm pore-size filters after prefiltration through 5.0 μm pore-size filters (diameter 47 mm; Whatmann, UK) (Niu et al. 2011; Rooney-Varga et al. 2005). The 5.0 μm filters were used to collect attached bacterial species, and 0.2 μm filters were used to collect free-living bacteria. The filters were immediately frozen in liquid nitrogen and stored at − 80 °C until processing.

### Chemical analysis

Composite samples were used to analyze the concentrations of total phosphorus (TP), dissolved total phosphorus (DTP), phosphate phosphorus (PO_4_-P), total nitrogen (TN), nitrate (NO_3_-N), ammonium (NH_4_-N), and chlorophyll *a* (Chl *a*) as described by Huang et al. (1999). PH, dissolved oxygen (DO), water temperature (T) and conductivity (COND) were measured 0.5 m below the water surface between 10 a.m. and 2 p.m. at each sampling site using a YSI ProPlus multiparameter water quality meter (Yellow Springs, OH, USA). The Secchi depth (SD) was measured using a black and white Secchi disk (20 cm diameter).

### Identification and phytoplankton counting

One liter water samples were preserved using 1% Lugol’s iodine solution and concentrated to 50 mL after settling for 48 h in Utermohl chambers and used to analyze phytoplankton composition (Huang et al. 1999). Concentrated samples (0.1 mL) were used for phytoplankton counting under 400× magnification using a light microscope (Olympus BX21, Tokyo, Japan) after mixing. Colonial *Microcystis* spp. cells were separated using an ultrasonic device (JY88-II, Scientiz, Ningbo, Zhejiang, China), and single cells of the colonies were counted. Taxonomic identification of the phytoplankton species was performed as described by Hu and Wei (2006). Phytoplankton counts revealed 46 clearly recognizable but rare species that were excluded from statistical analyses, as these species were either in very low numbers or found only on single sampling dates.

### Aquatic bacterial DNA extraction and 16S rRNA PCR and sequencing

Bacterial phylogenetic identities were assessed by PCR amplification and sequencing of the 16S rRNA gene. Total bacterial genomic DNA was extracted using the HiPure Water DNA Kit (Magen, Guangzhou, China) according to the manufacturer’s protocols. Total DNA was purified using a QIAGEN Plasmid Mega Kit (25) (QIAGEN, Valencia, CA, USA). Total DNA samples were characterized by 2.0% agarose gel electrophoresis. Extracted DNA was stored at − 80 °C prior to use for template analyses. Bacterial 16S rRNA genes were amplified using the following universal primers: barcodes 341F (CCTAYGGGRBGCASCAG) and 806R (GGACTACNNGGGTATCTAAT). Primers were also designed to amplify the V3 and V4 regions (~ 466 bp) of the 16S rRNA gene (Yu et al. 2005). PCR (ETC811, Beijing, China) kits (50 μL) contained 5 μL of 10 × KOD buffer, 3 μL of 25 mM MgSO_4_, 5 μL of each 2 mM dNTPs, 1.5 μL of each primer, 1 μL of KOD-plus, and 100–300 ng of template DNA. PCR cycling parameters included a 2 min initial denaturation at 94 °C followed by a thermal cycling program as follows: 2 min initial denaturation at 94 °C, 10 s denaturation at 98 °C, 30 s annealing at 62 °C, and a 30 s extension at 68 °C for 30 cycles, followed by a final 5 min extension at 68 °C. Amplicons were pooled, purified and quantified using the ABI Stepone Real-Time PCR System (Thermo Scientific, USA). Next-generation sequencing (NGS) was performed using the Illumina Hi-Seq 2500 PE250, which was operated by Genedenovo Inc. (Guangzhou, China).

### Statistical analyses

Sequences (excluding cyanobacterial sequences) were grouped into operational taxonomic units (OTUs) with similarities ≥ 97%. To test the initial hypothesis, heatmap correlations were generated amongst the phytoplankton genera, bacterial OTUs, and abiotic environmental parameters using linear regression (α = 0.05). Dominant bacterial OTUs that contained > 85% of the total sequences were selected. Dominant phytoplankton genera containing > 95% of the total cell density were selected. To test the second hypothesis, variation partitioning of the response variables was employed (i.e., bacterial the community compositions at an OTU resolution) to examine the relationships between phytoplankton genera and environmental parameters. The statistical approaches included variation partitioning (Anderson and Cribble 1998) in redundancy analysis (Rao 1964) and linear regression with multiple variables based on Mantel tests (Mantel 1967). To ensure the community data suited the requirements of linear ordination methods, we used Hellinger-transformed abundance data (Legendre and Gallagher 2001). We present only adjusted R^2^ values of our models. The ‘vegan’ package in R was used to run the variation partitioning analyses (Oksanen et al. 2013). We used a forward selection method developed by Blanchet et al. (2008) to select explanatory variables (i.e., the taxonomy of the phytoplankton and environmental variables) for final statistical analyses, thereby preventing exaggeration of the explanatory power of our constrained ordination models. We performed an ANOVA-like permutation test for redundancy analysis to assess the significance of constraints. We used the function ‘ordiR2step’ in the ‘vegan’ package in R to select explanatory variables (Oksanen et al. 2013). Identical methods were then used to examine the relationships amongst attached bacterial OTUs and free-living bacterial OTUs, abiotic environmental parameters and phytoplankton genera, the manner in which the free-living bacterial OTUs were influenced by the attached bacterial OTUs, abiotic environmental parameters and phytoplankton genera. All sequences used in the study are publicly available at the NCBI Sequence Read Archive (http://www.ncbi.nlm.nih.gov/Traces/sra) under accession IDs PRJNA488008 and PRJNA487989.

## Results

### Abiotic environmental parameters

The nutrient concentrations (TN, TP) and physical parameters [Secchi depth (SD), water temperature (T), dissolved oxygen (DO), conductivity (COND), pH and oxidation–reduction potential (ORP)] of the two sampling sites are shown in Additional file 1: Figure S1. The average TN of the two sites was 0.69 mg/L. The average TP of the two sites was 0.03 mg/L. The highest concentrations of TN at Site 1 (Additional file 1: Figure S1a) and Site 2 (Additional file 1: Figure S1b) occurred on October 2014, whilst the maximum TP at Site 1 (Additional file 1: Figure S1a) and Site 2 (Additional file 1: Figure S1b) occurred during August 2013 and May 2013, respectively.

### Phytoplankton successions

**Fig. 2:**
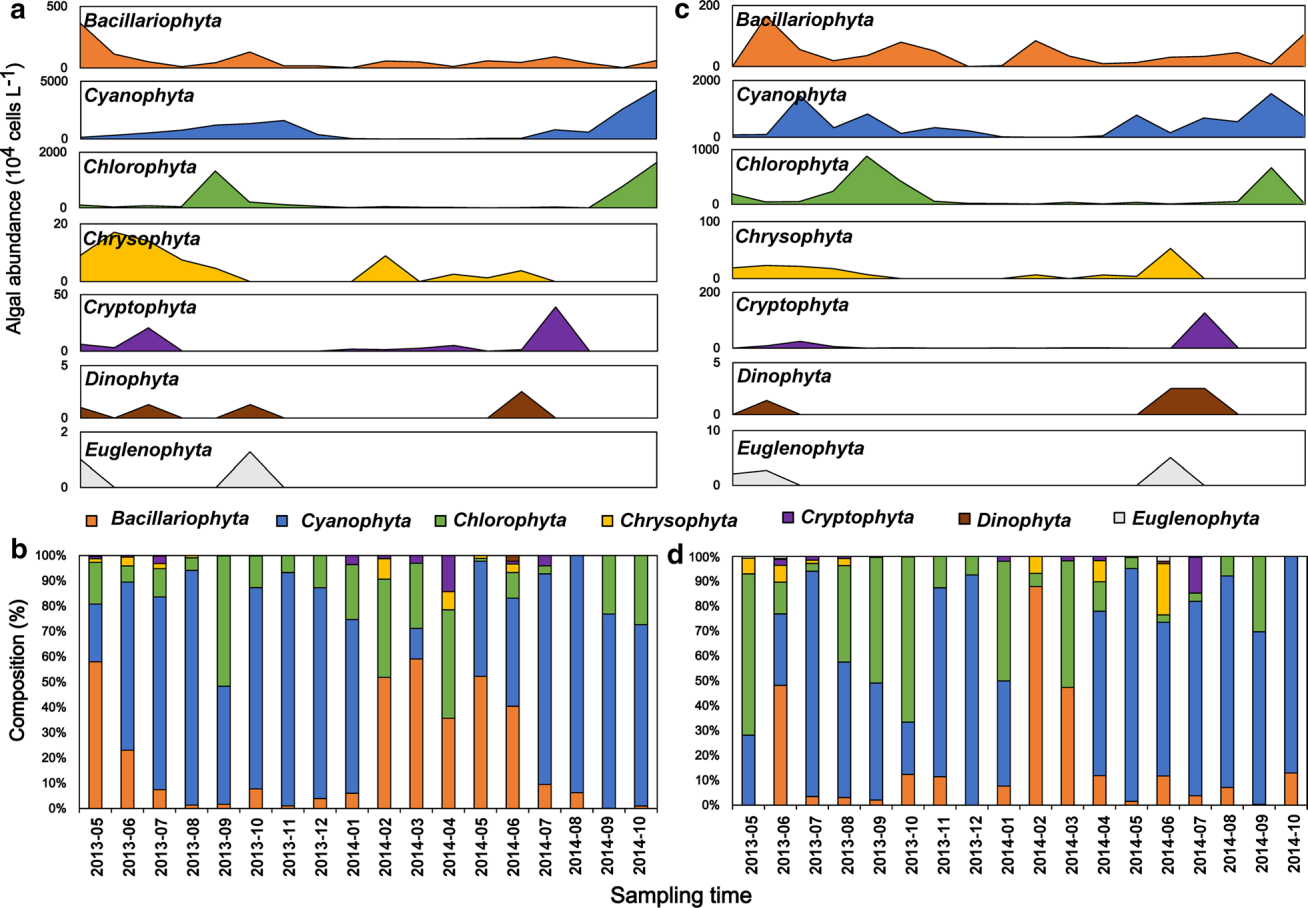
Seasonal variations in the phytoplankton phyla in Lake Erhai from May 2013 to October 2014 at the two sampling sites (**a**, **b** for site 1; **c**, **d** for site 2). **a**, **c** Densities of the phytoplankton phyla; **b**, **d** compositions of the phytoplankton phyla. Color codes represent the same phyla in each panel, and the Y-axis scales differ

The sampling campaign lasted 18 months and included two cyanobacterial blooms. The successional patterns in the phytoplankton community composition during the sampling period (May 2013 to October 2014) are shown in Fig. 2. We quantified 55 phytoplankton genera (27 *Chlorophyta*, 9 *Bacillariophyceae*, 8 *Cyanophyceae*, 4 *Chrysophyceae*, 3 *Euglenophyceae*, 2 *Dinophyceae* and 2 *Cryptophyta*). *Cyanophyta*, *Chlorophyta* and *Bacillariophyta* were the dominant phyla at both sites (the compositions of *Cyanophyta*, *Chlorophyta* and *Bacillariophyta* at Site 1: 67%, 23% and 7%; Site 2: 72%, 22% and 6%, respectively) (Fig. 2). *Cyanophyta* was the dominant phylum from July to December 2013 and from May to October 2014 (cyanobacterial bloom period), whilst *Bacillariophyta* dominated from February to June 2014 (diatom-dominated period). *Chlorophyta* was dominant in the summer and decreased in numbers from summer to winter.

Cyanobacterial blooms in Lake Erhai occurred during the summer and autumn when the water temperatures exceeded 13 °C (Fig. 2). The counts varied amongst the dominant groups (Additional file 1: Figure S2). The nine dominant algal genera (> 95% of the total cell density at the two sites are shown (Additional file 1: Figure S2). *Microcystis* was the dominant genus in the phylum *Cyanophyta* (cell density > 60% at both sites) and *Aphanizomenon* and *Anabaena* occurred at high water temperatures. The abundance of *Fragilaria* and *Melosira* were maximal during the winter and spring. Both sites had varying cell densities of *Microcystis* during the sampling period (Additional file 1: Figure S2). In addition, *Chlorophyta Psephonema* dominated during *Microcystis* blooms, particularly in September at both sites (Additional file 1: Figure S2).

### Composition of the bacterial communities

High-throughput sequencing of the 16S rRNA genes reflected the seasonal succession in the bacterial communities; a total of 273 bacterial families from the attached and free-living communities affiliated with nine bacterial phyla were determined. Both the free-living and attached bacterial communities harbored similar dominant phyla composed of *Proteobacteria* (23 families), *Bacteroidetes* (16 families), *Actinobacteria* (56 families) and *Firmicutes* (32 families), but their proportions changed seasonally (Additional file 1: Figure S3). The attached bacterial community was composed of *Proteobacteria* (Site 1: 56%; Site 2: 56%), *Firmicutes* (Site 1: 15%; Site 2: 24%), *Bacteroidetes* (Site 1: 13%; Site 2: 9%), and *Actinobacteria* (Site 1: 10%; Site 2: 7%) (Additional file 1: Figure S3a, c), while the free-living bacteria had the same dominant phyla: *Actinobacteria* (Site 1: 54%; Site 2: 44%), *Proteobacteria* (Site 1: 35%; Site 2: 27%), *Firmicutes* (Site 1: 4%; Site 2: 22%), and *Bacteroidetes* (Site 1: 4%; Site 2: 5%) (Additional file 1: Figure S3b, d).

### Effects of abiotic environmental factors and phytoplankton on attached and free-living bacteria

**Fig. 3:**
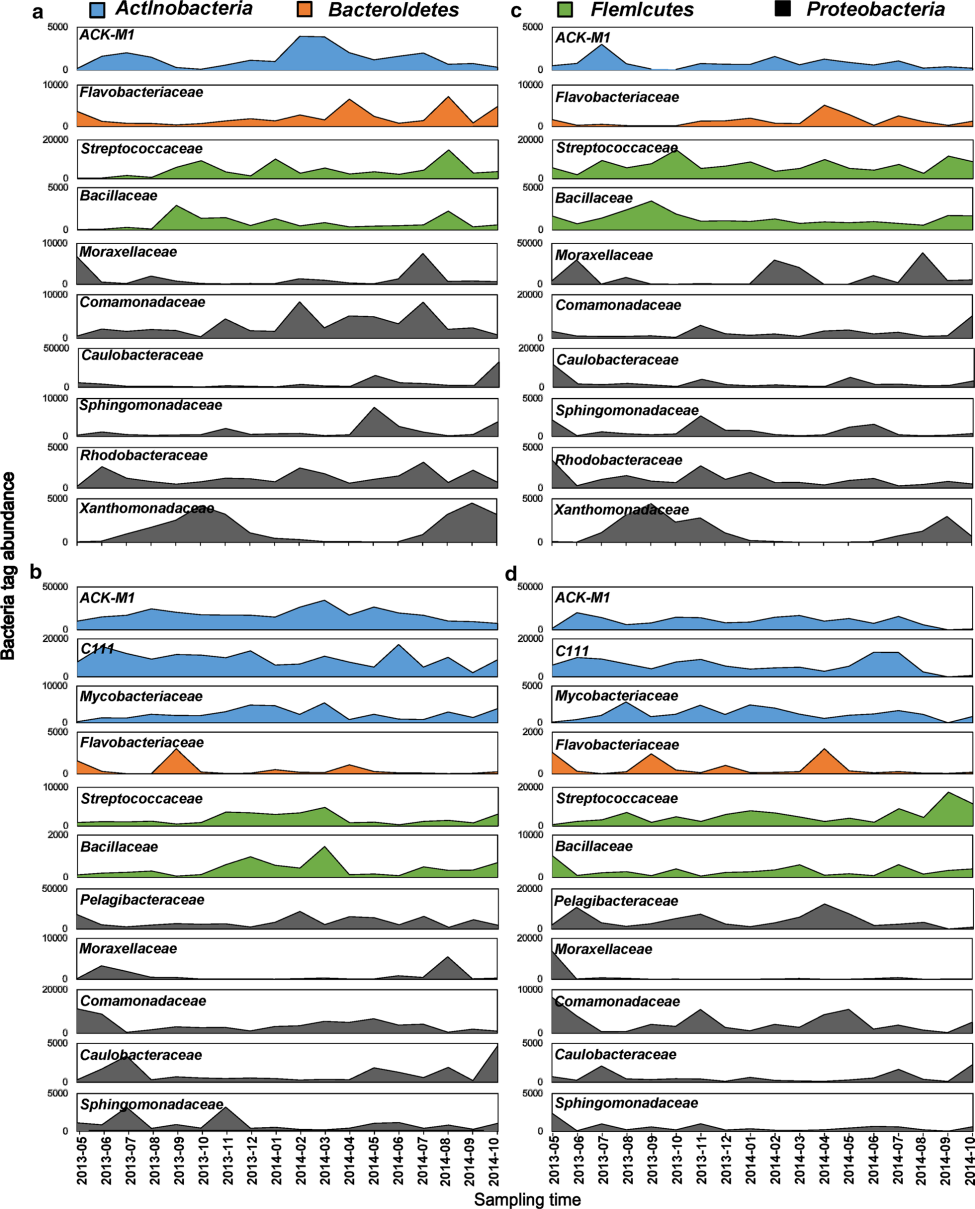
Seasonal variations in the top 10 dominant bacterial families in Lake Erhai from May 2013 to October 2014 at the two sampling sites (**a**, **b** for site 1; **c**, **d** for site 2). **a**, **c** Composition of the 10 dominant attached bacterial families; **b**, **d** composition of the dominant free-living bacterial families. Color codes represent the same phyla in all pictures, and the Y-axis scales differ

**Fig. 4:**
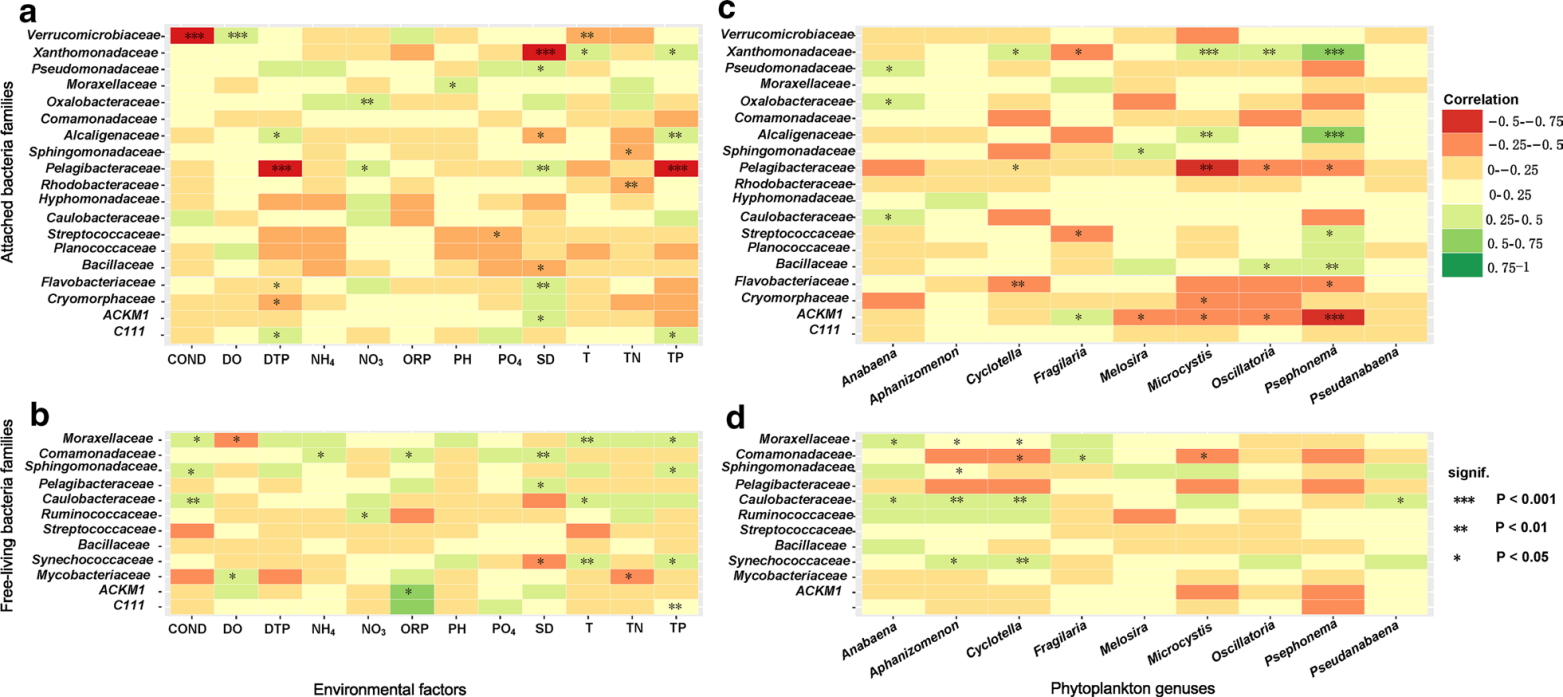
Correlation heatmap amongst dominant phytoplankton genera, bacterial families and environmental parameters. **a** Attached bacterial families and environmental parameters; **b** free-living bacterial families and environmental parameters; **c** attached bacterial families and phytoplankton genera; **d** free-living bacterial families and phytoplankton genera. Different colors and spots represent shifted species associations. For clarity, connections between the remaining species are not displayed (starred numbers show the degree of significance; different colors show the different degrees of positive and negative connections)

The heatmap (Fig. 4) shows the correlation amongst the dominant phytoplankton genera, bacterial families and abiotic environmental parameters. Figure 3a and c show that the attached bacterial communities were dominated by three families: *Firmicutes Streptococcaceae*, *Proteobacteria Moraxellaceae* and *Proteobacteria Rhodobacteraceae.* In addition, the four endemic dominant families included the *Bacteroidetes Flavobacteriaceae*, *Betaproteobacteria Alcaligenaceae*, *Proteobacteria Rhodobacteraceae* and *Xanthomonadaceae*. Members of the *Streptococcaceae* group dominated the attached bacterial community throughout the year. *Moraxellaceae* and *Rhodobacteraceae* showed no significant correlation with algae, but members of the *Streptococcaceae* group positively correlated with *Chlorophyta Psephonema* (*p *< 0.05) and negatively correlated with PO_4_-P (*p* < 0.05) (Fig. 4). *Moraxellaceae* positively correlated with the pH (*p* < 0.05). With the onset of summer, the relative abundance of *Proteobacteria* increased, followed by *Xanthomonadaceae*, *Betaproteobacteria* and *Alcaligenaceae*. As soon as the *Cyanophyta Microcystis* and the *Chlorophyta Psephonema* increased (bloom phase), *Xanthomonadaceae* (*Proteobacteria*) and *Alcaligenaceae* (*Betaproteobacteria*) showed steep increases in numbers (Fig. 3). The heatmaps (Fig. 4) showed highly significant correlations of the bloom algae (*Microcystis* and *Psephonema*) and positive correlations between *Xanthomonadaceae* and *Microcystis* (*p* < 0.001), *Xanthomonadaceae* and *Psephonema* (p < 0.001), *Alcaligenaceae* and *Microcystis* (*p* < 0.01), and *Alcaligenaceae* and *Psephonema* (*p* < 0.001). The *Rhodobacteraceae* negatively correlated with the TN (*p* < 0.05). *Flavobacteriaceae* positively correlated with the SD (*p* < 0.05). Figure 3b, d show that the free-living bacterial communities were dominated by the top four families of *Actinobacteriage ACK*-*M1* and *C111*, the *Proteobacteria Pelagibacteraceae* and *Firmicutes Streptococcaceae*. In addition, *C111, Actinobacteria Mycobacteriaceae* and *Pelagibacteraceae* were the three endemic families that dominated the free-living bacteria. Figure 4 shows a significant positive correlation between *ACK*-*M1* and ORP (*p *< 0.05), and a significant negative correlation between *C111* and TP (*p* < 0.01). *Pelagibacteraceae* positively correlated with the SD (*p* < 0.05). *Mycobacteriaceae* positively correlated with the DO (*p* < 0.05) but negatively correlated with the TN (*p* < 0.05). *ACK*-*M1, C111, Mycobacteriaceae, Pelagibacteraceae* and *Streptococcaceae* showed no significant positive correlations with the algae.

**Fig. 5:**
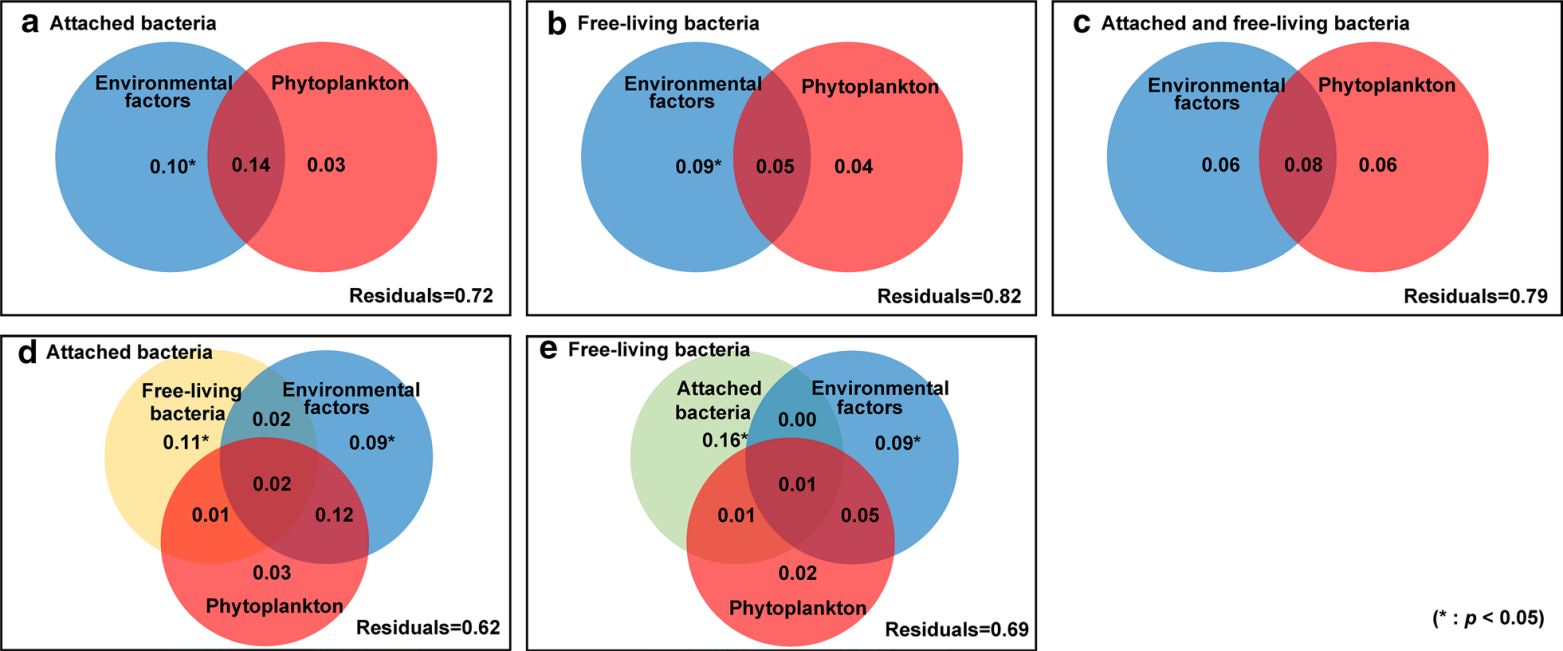
Variation partitioning in redundancy analysis ordination of phytoplankton genera, bacterial families, and environmental parameters. **a** Attached bacterial families with environmental parameters and phytoplankton genera; **b** free-living bacterial families with environmental parameters and phytoplankton genera; **c** bacterial families with environmental parameters and phytoplankton genera; **d** attached bacterial families with free-living bacterial families, environmental parameters and phytoplankton genera; **e** free-living bacterial families with attached bacterial families, environmental parameters and phytoplankton genera. (Starred numbers indicate the level of significance)

Variation partitioning of the phytoplankton genera and abiotic environmental parameters showed scale-dependent processes that structured attached and free-living bacterial communities (Fig. 5). Abiotic environmental parameters had higher and more significant association with changes in the bacterial community compared to the phytoplankton community. For the attached bacteria, independently described abiotic environmental variations (10%) were higher than independent phytoplankton variations (3%), and the shared fraction explained part of the total variation (14%) (Fig. 5a). Moreover, for free-living bacteria, the effects of abiotic environmental changes explained 9% of the variation, alterations in the phytoplankton species composition explained 4%, and the shared fraction explained 5% of the total variation (Fig. 5b). For total bacteria, the coupled effects of the abiotic environmental and phytoplankton variations described 8% of the variation, whilst abiotic environmental factors and phytoplankton variations explained only 6% of the total variation (Fig. 5c). We analyzed the relationships amongst the attached bacteria, free-living bacteria, phytoplankton genera and abiotic environmental parameters (Fig. 5d, e). Variation partitioning analysis showed that the free-living bacteria independently described 11% of the variations in the attached bacterial community composition, abiotic environmental factors explained 9% of the variation, whilst phytoplankton variations explained 3% (Fig. 5d). The variation partitioning analysis showed that the attached bacteria accounted for 16% of the variation in the free-living bacterial community, environmental factors accounted for 9% of the variation, and phytoplankton accounted for 2% of the variation (Fig. 5e). Taken together, the species composition of the bacterial community (either free-living or attached) changed not only with the species composition of the phytoplankton community, but also with abiotic environmental factors which were the dominant influence. The species composition of the attached bacterial communities were shaped by a combination of free-living bacteria, abiotic environmental factors and phytoplankton, but the phytoplankton affected only several rare families, particularly during bloom periods (*Xanthomonadaceae* and *Alcaligenaceae*).

## Discussion

In this study, the relationships amongst the free-living and attached bacterial community compositions, phytoplankton species composition and abiotic environmental factors in Lake Erhai were investigated using partitioning analysis. The results revealed that the abiotic environmental had a greater effect than phytoplankton succession on both free-living and attached bacterial compositions, even though the attached bacteria were strongly associated with the phytoplankton community during specific periods. The impact of environmental factors on both free-living and attached bacterial community compositions were greater than those of the phytoplankton community, amongst which the TP, SD, water temperature, DO and COND strongly affected bacterial community composition. The *Microcystis* bloom and the sub-dominance of *Psephonema* resulted in species-specific bacteria (families *Xanthomonadaceae* and *Alcaligenaceae*) that dominated the attached bacterial communities. Recent studies highlight how the power of variation partitioning analysis can vary depending on the quality of the data (Jones et al. 2008) and the spatial and temporal nature of the study (Mykrä et al. 2010). This is due to the classical forward selection methods in regression or canonical redundancy analysis presenting a highly inflated Type I errors and overestimates of the variances (Legendre et al. 2009). In this study, the high residual variation partitioning analysis (the low power of the statistics) were due to a low number of explanatory variables in seasonal site scales, which can be improved by utilizing more explanatory variables based on the current knowledge of microbial environments across different scales.

### Bacterial community compositions are influenced by abiotic and biotic factors

The diversity, abundance and composition of the bacterial communities in aquatic ecosystems are prone to environmental fluctuations and can be strongly affected by physicochemical environmental factors, including salinity, latitude, PO_4_, light, dissolved organic carbon (DOC), T, pH, TN, TP and DO (Bachmann et al. 2018; Fraser et al. 2018; Kirchman et al. 2017; Luria et al. 2017; Paver and Kent 2017; Romina Schiaffino et al. 2011). However, as a major contributor to the primary productivity in eutrophic lakes, the phytoplankton plays important roles in shaping bacterial community composition (Berry et al. 2017; Goecke et al. 2013; Landa et al. 2016; Luria et al. 2017). Furthermore, several studies demonstrate that the effects of phytoplankton species composition on bacterial communities is closely related to DOC exudates from the phytoplankton, and this indirect effect temporally lags behind the algal bloom (Landa et al. 2016; Luria et al. 2017). Our previous studies also showed that the DOC is an important contributor to bacterial community compositions in Lake Taihu (Pang et al. 2014). As a result, the influence of phytoplankton succession on the bacterial community may be underestimated when monthly scale sampling is used. In future studies, more frequent sampling will provide further insight into the relationship between DOC exudates and bacterial species composition. In this study, both the physicochemical environmental factors and phytoplankton communities explained the variations in bacterial community composition in Lake Erhai. Similar results were observed in other lakes, showing that the combined effects of the abiotic environmental factors and phytoplankton species composition regulated the abundance and composition of the bacterial communities (Table 1), implying that physicochemical factors, such as Chl *a*, phytoplankton biomass or phytoplankton structures, are capable of changing the bacterial community composition. However, quantification of the relative level of influence of each factor on the bacterial community composition was not investigated in the studies.

**Table 1:** Coupled effects of environmental factors and phytoplankton communities on the bacterial communities of the water columns

System	Response variables	Predicted variables	References
Five lakes, Sweden	Bacterial abundance (DGGE band number)	Nutrient content of the lakes, biomasses of microzooplankton, cryptophytes and chrysophytes	Lindström (2000)
Five mesotrophic lakes, Sweden	Bacterial abundance (DGGE band number)	T, diatom biomass, and cryptophyte biomass	Lindström (2001)
Lake Toolik, Alaska	Bacterial abundance (16S rRNA gene sequences of bacteria)	DOC (released by phytoplankton)	Crump et al. (2003)
Thirteen lakes in Wisconsin, USA	Bacterial abundance (ARISA fragment richness)	DOC, Chl *a* and WT	Yannarell and Triplett (2004)
Six north temperate humic lakes in Wisconsin, USA	Bacterial abundance (ARISA fragment richness)	Meteorological, environmental and biological data set	Kent et al. (2007)
Thirty-five rock pools at the Baltic Sea coast, Sweden	Bacterial abundance (T-RFLP data)	Spatial variables, salinity, Chl *a*, and water color	Langenheder and Ragnarsson (2007)
Lake Taihu, China	Bacterial abundance (16S rRNA gene sequences of the bacteria)	Biomass of phytoplankton and WT	Niu et al. (2011)
Lake Taihu, China	Bacteria diversity (Shannon) and species richness (DGGE band number)	The similarities of *Microcystis*-associated, settling particle-associated and free-living bacteria	Shi et al. (2012)
Lake Erie, USA	Bacterial abundance (OTU richness)	Chl *a,* pH, temperature	Berry et al. (2017)
Two north temperate humic lakes in Vilas County, Wisconsin	Bacterial diversity (Bray–Curtis similarities)	Light, temperature, and phytoplankton	Paver and Kent (2017)

### Abiotic environmental factors override phytoplankton succession in shaping the bacterial community

Abiotic environmental factors have a direct and indirect influence on seasonal variations in the abundance and composition of bacterial communities. Nutrient concentrations and ratios, in addition to other abiotic environmental factors, are crucial for bacterial succession (Fraser et al. 2018; Kirchman et al. 2017; Paver and Kent 2017; Tang et al. 2017). Bacterial biomass positively correlates with the trophic status of the lakes (Scofield et al. 2015) but does not correlate with the chlorophyll level (Adamovich et al. 2019). Abiotic factors invariably serve as restriction factors for bacterial communities as each bacterial taxon has optimal, minimal and maximal environmental conditions (temperature, TP and TN concentration) (Scofield et al. 2015), and fluctuations in the abiotic environmental conditions result in a succession of bacteria (Luria et al. 2017; Paver and Kent 2017). Succession in the phytoplankton community is mediated by abiotic environmental factors, which likely contribute to variations in the bacterial community composition. We therefore used variation partitioning analyses to determine whether the bacterial communities were independently related to changes in the phytoplankton composition and abiotic environment. Of particular interest was that the top four dominant families of the free-living bacteria (*ACK*-*M1, C111, Pelagibacteraceae* and *Streptococcaceae*) significantly correlated with physicochemical environmental factors, but did not correlate with the algal community composition.

### Phytoplankton community composition is related to species-specific bacterial communities

Although phytoplankton species composition plays a key role in regulating the bacterial community composition in experimental and natural systems (Berry et al. 2017; Camarena-Gómez et al. 2018; Gomez et al. 2019; Kirchman et al. 2017), this study suggested that succession in the phytoplankton community plays an important role in influencing attached and free-living bacterial communities during specific periods. We found that phytoplankton significantly influenced the dynamics of bacterial communities only when *Microcystis* and *Psephonema* rapidly increased rapidly during the bloom period. This could be because closely related phytoplankton taxa produce similar metabolic products (Jasti et al. 2005; Rooney-Varga et al. 2005) and subsequently support similar bacterial communities. We verified that differences in the phytoplankton species composition support the development of specific bacterial families during different phases (Dai et al. 2016; Tang et al. 2017). The families *Xanthomonadaceae* and *Alcaligenaceae* showed steep increases in numbers as the density of *Microcystis* and *Psephonema* increased. The families *Xanthomonadaceae* and *Alcaligenaceae* were be considered species-specific bacteria for *Microcystis* and *Psephonema* in Lake Erhai. The highly significant positive correlation between dominant algae and their species-specific bacteria in aquatic ecosystems suggests an important relationship between algal and bacterial communities (Niu et al. 2011; Shi et al. 2012). Our results show that the variation in bacterial community composition correlated with phytoplankton succession, particularly the variation in attached bacterial communities.

### Attached and free-living bacterial communities

In this study, the rRNA genes from the attached bacteria were fundamentally homologous to those of free-living bacteria at the family classification level, suggesting that attached bacterial clones originate from the free-living bacterial community. Riemann and Winding (2001) also suggested a significant phylogenetic overlap in free-living and particle-associated bacterial assemblages. Our data indicate that specific bacterial communities developed on attached bacteria, and these communities differed from those that dominated in free-living bacteria. However, almost all attached and free-living bacterial sequences were members of four distinct phyla: Proteobacteria, Bacteroidetes, Actinobacteria and Firmicutes. Several studies (Paver et al. 2013; Seymour et al. 2017) suggest that phytoplankton provide suitable microenvironments, which may explain why the dominant families of attached bacteria differ from those of free-living bacteria. Consequently, it is conceivable that the attached bacteria represent variations in the phylotype associated with the adaptation of free-living bacteria to the microenvironment and phytoplankton.

In this study, the relationships amongst free-living and attached bacterial community compositions, phytoplankton structures and abiotic environmental factors in Lake Erhai were explored through a detailed field survey. Variation partitioning analysis revealed that abiotic environmental factors had larger effects on both free-living and attached bacterial community compositions compared to phytoplankton succession even though the attached bacterial community composition was strongly associated with the phytoplankton community during specific periods. The *Microcystis* bloom plus *Psephonema* domination resulted in species-specific bacteria (families *Xanthomonadaceae* and *Alcaligenaceae*) that dominated the attached bacterial communities. In addition, the attached and free-living bacteria in freshwater ecosystems could interact, and mutually transform. Our results add a new understanding of microorganism and ecosystem functioning, even though more detailed studies are now required.

## Supplementary information

**Additional file 1.** Additional figures.

## Data Availability

All sequences are publicly available at the NCBI Sequence Read Archive (http://www.ncbi.nlm.nih.gov/Traces/sra) under accession IDs PRJNA488008 and PRJNA487989.

## Abbreviations

TP: total phosphorus
DTP: dissolved total phosphorus
PO_4_-P: phosphate phosphorus
TN: total nitrogen
NO_3_-N: nitrate
NH_4_-N: ammonium
Chl *a*: chlorophyll *a*
DO: dissolved oxygen
T: water temperature
COND: conductivity
SD: Secchi depth
DOC: dissolved organic carbon
