# Supplementary material for: Abiotic environmental factors override phytoplankton succession in shaping both free-living and attached bacterial communities in a highland lake
Source: AMB Express. 2019 Oct 31;9:170. doi: 10.1186/s13568-019-0889-z (PMC6823470; doi:10.1186/s13568-019-0889-z)
Supplement: Supplementary file 1 — Additional file 1. Additional figures. [file 13568_2019_889_MOESM1_ESM.docx]

## Additional information


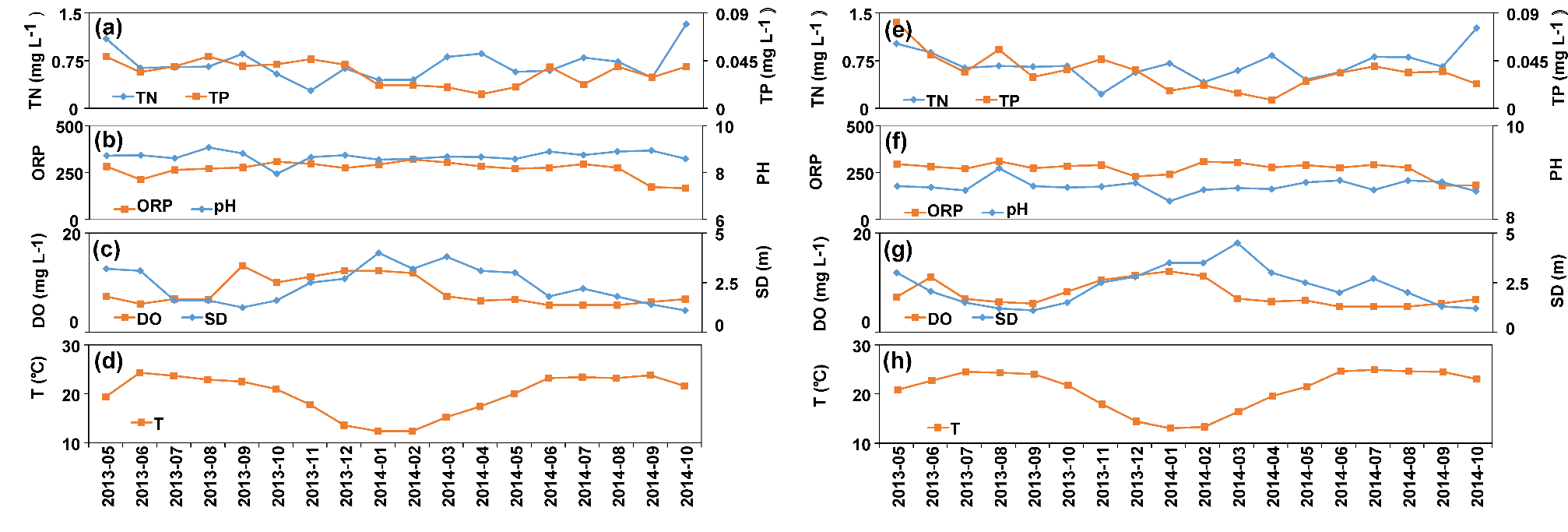


Figure S1. Temporal variations in the abiotic parameters in Lake Erhai from May 2013 to October 2014 at the two sampling sites (a, b, c and d for site 1; e, f, g and h for site 2). (a) and (e) TN and TP; (b) and (f) ORP and pH; (c) and (g) DO and SD; (d) and (h) T. Note the different scales of the Y-axis.


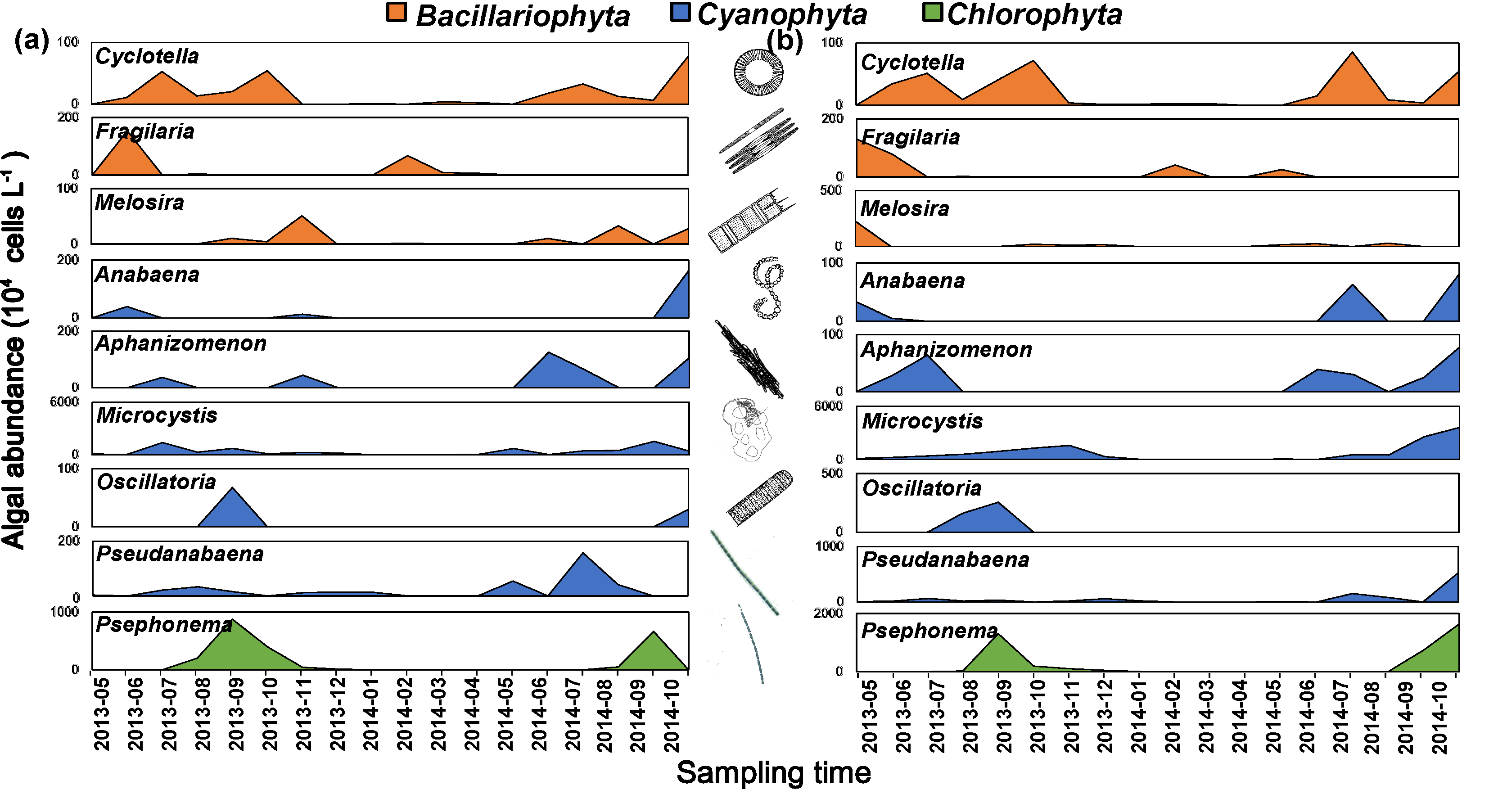


Figure S2. Abundance (10^4^ cells L^-1^) of the dominant phytoplankton genera in Lake Erhai from May 2013 to October 2014 at the two sampling sites (a for site 1; b for site 2). Color codes are the same for the phyla in all images Y-axis scales differ. Images are from freshwater microorganisms (Zhou, F., and J. Chen. 2005. Freshwater microorganism images Chemical Industry Press) and microscopic imaging.


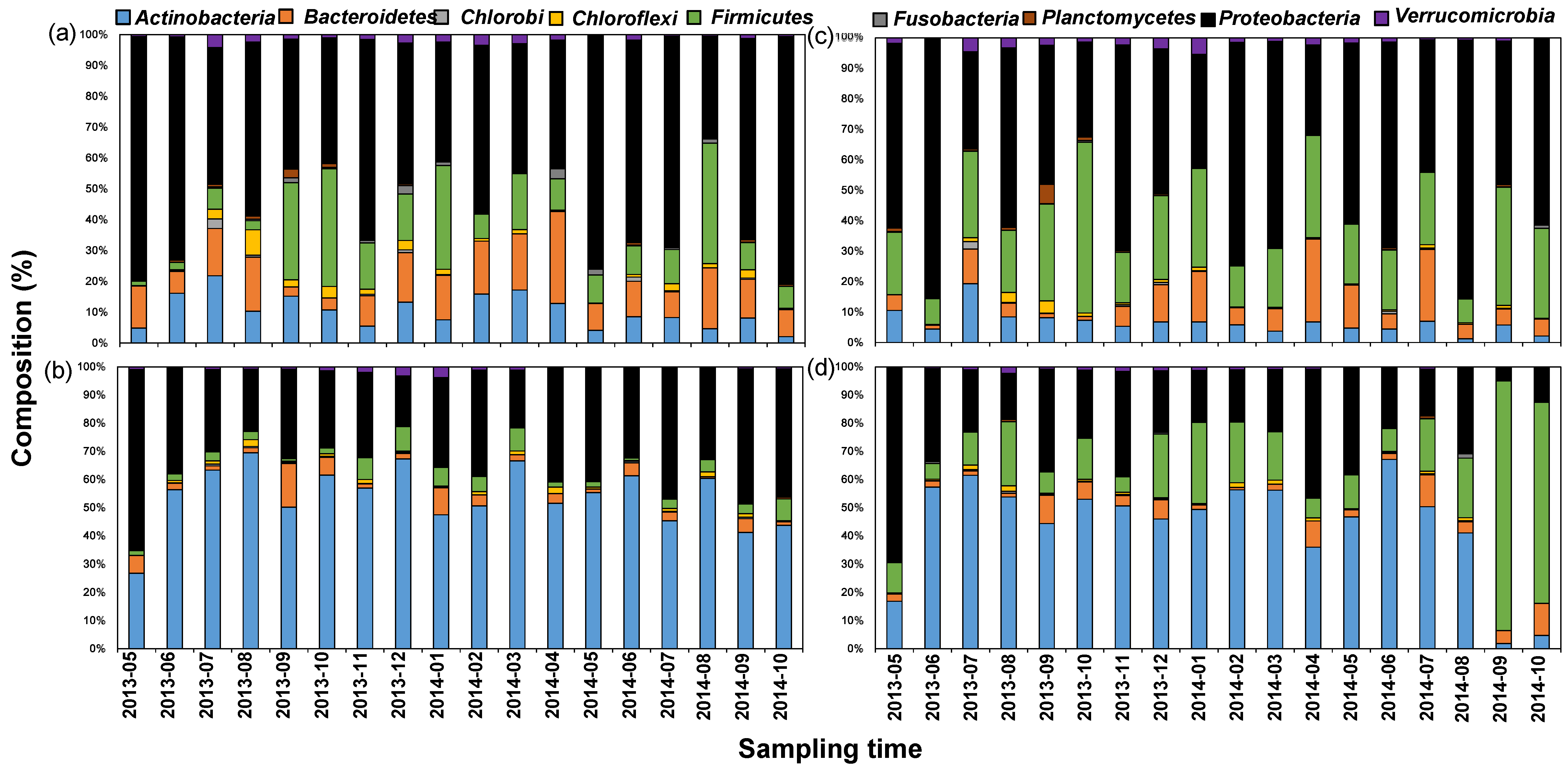


Figure S3. Seasonal variations in the dominant bacterial phyla in Lake Erhai from May 2013 to October 2014 at the two sampling sites (a and b for site 1; c and d for site 2). (a) and (c) composition of the dominant attached bacterial phyla; (b) and (d) composition of the dominant free-living bacterial phyla. Color codes are the same for the phyla in each panel.
